# Psoriasis risk allele function in activated Th1/17 cells with “memory” to antigen exposure

**DOI:** 10.1371/journal.pone.0344675

**Published:** 2026-03-11

**Authors:** Bayazit Yunusbayev, Sergei Ryakhovsky, Radick Altinbaev, Anastasia Kislova, Kseniya Danilko, Liudmila Kraeva, Milyausha Yunusbaeva

**Affiliations:** 1 Institute of Translational Biomedicine, St Petersburg State University, Saint-Petersburg, Russia; 2 Department of Genetics and Biotechnology, St Petersburg State University, Saint-Petersburg, Russia; 3 SCAMT Institute, ITMO University, Saint-Petersburg, Russia; 4 Laboratory of Neurophysiology of Learning, Institute of Higher Nervous Activity and Neurophysiology of RAS, Moscow, Russia; 5 Cell Culture Laboratory, Bashkir State Medical University, Ufa, Russia; 6 Laboratory of Medical Bacteriology, Saint-Petersburg Pasteur Institute, Saint-Petersburg, Russia; 7 Laboratory of Functional Genomics, Saint-Petersburg State Phthisiopulmonology Research Institute, Saint-Petersburg, Russia; West Bengal University of Animal and Fishery Sciences, INDIA

## Abstract

Most causal variants for complex diseases are expected to affect gene regulation in a cell- and context-specific manner. Hence, identification of such dynamically functioning variants requires functional readouts in disease-relevant tissues and context. In this study, we prioritized causal variants for psoriasis by adding functional annotations from disease-relevant cells. We demonstrate that disease-relevant immune cells, unlike most other tissues, possess functional annotations that match candidate causal SNPs. Specifically, we identified an eQTL, rs4672505, that reduces *B3GNT2* gene expression only in Th1/Th17 cells with a memory phenotype, i.e., antigen-experienced T helper cells. This eQTL, a likely causal variant, also matched an enhancer chromatin mark exclusive to memory T helper cells and absent in other tissues. A disease-risk allele A at the eQTL correlates with reduced expression of the B3GNT2 glycosyltransferase. B3GNT2 deficiency in murine models reduces the glycosylation of the CD28 co-receptor involved in the CD28/B7 co-stimulation pathway and results in increased T cell activation upon antigen stimulation. We hypothesize that the risk allele A in patients increases the activation of memory Th1/Th17 cells upon re-exposure to antigens, which constitutes “signal 1”. Increased reactivity to antigens depends on “signal 2” via CD28/B7 co-stimulation from antigen-presenting cells that need to encounter microbial products. Hence, this genetic risk mechanism lies at the nexus of the response to specific antigens and microbial exposure, for instance, infection or vaccination, both of which are known to exacerbate psoriasis.

## Introduction

Most causal variants (~90%) for inflammatory diseases are expected to be non-coding and affect disease risk through gene regulation [1] and splicing [2]. For non-coding variants, traditional challenges of fine-mapping, such as strong linkage disequilibrium (LD), are further complicated by the difficulty in identifying the relevant cell type and effector gene that mediates function [3]. This is because non-coding variants contribute to disease by affecting different stages of gene regulation and splicing [2]. Gene regulation is dynamic, often in unknown cells and specific physiological contexts [4]. In autoimmune diseases, stimulus-dependent regulatory SNPs specific to immune cells show greater overlap with putative risk SNPs [5–7]. This is likely also true for psoriasis, for which dozens of genome-wide association studies (GWAS) have identified nearly 100 risk loci; however, so far, only a few have been fine-mapped and studied functionally [8]. While fine-mapping can be improved by seeking functional SNPs [9], popular resources, such as The Genotype-Tissue Expression (GTEx) [10], The Encyclopedia of DNA Elements (ENCODE) [11], and NIH Roadmap Epigenomics (ROADMAP) [12], represent only a fraction of the known functional variants in the human genome, derived from healthy donor tissues or tissues from select disorders. Therefore, to achieve functionally-informed mapping, it is desirable to have functional genomic annotations tailored to the disease in focus, along with relevant immune cell types and physiological states, such as stimulation by microbial products.

Since most causal SNPs are non-coding, predicting their effector gene is not straightforward, the “gene for which the product is predicted to mediate the effect of a genetically associated variant on a disease” [3]. Previous studies have nominated effector genes for psoriasis risk loci, initially using the nearest gene approach and more recently employing a transcriptome-wide association study (TWAS) [8,13–15]. Nevertheless, true effector genes can be missed, even with functionally-informed fine-mapping tools, if relying on genomic annotations from standard tissues not relevant to disease. To increase fine-mapping success, we considered a wide range of cells and tissues and maximized functional annotations from primary immune cells pertinent to disease [16,17]. Specifically, we expanded the standard eQTL data set from 48 GTEx tissues [10,18] with eQTLs from 23 immune cells and states published elsewhere [18–22]. In doing so, we avoided functional readouts from cells with perturbed genetic and epigenetic landscapes, such as immortalized cell lines or induced pluripotent stem cells (iPS cells). We paid special attention to the fact that psoriasis, like other autoimmune diseases, can be triggered by exposure to infections [23,24]. Therefore, in this study, we assumed that some of the missing psoriasis-associated causal variants manifest as regulatory variants in response to immune-cell-activating stimuli, such as infection, vaccine, or other sources of danger signals. Based on this working hypothesis, we aimed to fine-map known psoriasis risk loci for which previous studies did not identify functional SNPs in standard tissues and cells under steady-state conditions. To achieve this goal, we sought to add functional annotations for immune cells that were activated, challenged by diverse microbial ligands, or had memory of prior antigen exposure.

## Materials and methods

### GWAS dataset and statistical fine-mapping

To carry out genetic fine-mapping for psoriasis risk loci, we used GWAS summary statistics for 9 010 555 SNPs published earlier [15]. The GWAS summary statistics were obtained from the NHGRI-EBI Catalog https://www.ebi.ac.uk/gwas using the study identifier GCST90019016. To organize candidate SNPs around established psoriasis risk loci, we used 64 index SNPs reported in S5 Table in the original study [15] (S1 Table). Before fine-mapping, we performed quality control (QC) and harmonization using the MungeSumstats R package [25]. Altogether, 7 791 492 SNPs were retained for downstream analyses after QC and harmonization. To carry out fine-mapping, we integrated summary statistics with the linkage disequilibrium (LD) data from a reference population of European ancestry (EUR) in the 1000 Genomes project phase 3 [26]. A statistical fine-mapping step was performed for each locus separately with susieR [27] using the echolocatoR pipeline [28]. For each locus, we assumed a maximum of five causal SNPs and ran fine-mapping using variable genomic window sizes (30 kb, 60 kb, 150 kb, 250 kb, and 450 kb). We chose the window size that best captured the LD structure around the lead SNP. For each locus, fine-mapping inferred the posterior probability (PP) of each SNP being causal. Depending on the local distribution of SNP posterior probabilities, we defined three fine-mapping outcomes: a) One or more SNPs have strong evidence (PP ≥ 0.95) of being causal, b) one or more SNPs demonstrate higher PP of being causal than nearby SNPs, but with low probabilities (PP < 0.95) c) multiple SNPs are strongly linked and demonstrate similar but low PP of being causal.

### Colocalization of GWAS associations with eQTLs

The GWAS SNP coordinates for psoriasis were lifted to the GRCh38 reference genome using CrossMap [29] to match with the eQTL variant coordinates. We performed statistical colocalization of psoriasis GWAS SNPs with eQTLs obtained from the eQTL Catalogue [30], which stores uniformly standardized eQTL summary data from published sources. For colocalization, we used QTLs from 48 GTEx human body tissues [10,18] and 23 immune cell types and states (naïve, activated, and memory cells) [18–22]. While the eQTL Catalogue featured over 35 immune-related cells, we selected only 23 of them (S2 Table) by excluding reprogrammed iPS cells [20] and cells genotyped on microarrays [18]. The major problem with using eQTLs from iPS-reprogrammed cells is that these cells retain a residual epigenetic memory of the source somatic cells and exhibit de novo epigenetic aberrations [31]. To run colocalization analyses, we used the R package coloc (v.3.1) [32]. The P-value distributions for eQTL analyses were post-processed using the qvalue R package (version 2.28.076) to define the p-value threshold corresponding to a 10% false discovery rate (FDR). Co-localization between a QTL and a psoriasis SNP was considered significant if the target hypothesis provided strong evidence, as indicated by a posterior probability (PP4 ≥ 0.8). Here, PP4 denotes the posterior probability of hypothesis 4 when there is a shared SNP association between disease and eQTL. Colocalization analysis computes support (in terms of posterior probability) for all possible configurations (hypotheses), such as, for example, PP1 for hypothesis 1 – SNP association with disease, but not with eQTL, or PP3 for hypothesis 3 – SNP association with disease and eQTL, but there are two independent SNPs. For the total set of 7 791 492 GWAS SNPs, we found 194 eQTL-tissue pairs with PP4 ≥ 0.8 at 10% FDR (S3 Table). For our analyses, we retained only 39 colocalized eQTLs that fell within one of the 64 established psoriasis risk loci (S4 Table). Specifically, eQTLs were retained if they were in strong LD with one of the lead SNPs from 64 risk loci.

### Overlap with the ROADMAP chromatin states associated with regulatory elements

To identify overlaps between colocalized eQTLs and regulatory elements, we used chromatin-state annotations from the ROADMAP project [12]. We analyzed chromatin states within genomic windows containing our eQTL of interest and the affected genes. In doing so, we first explored chromatin states in the same cell type in which the focal eQTL was identified. We next tested whether our cell-specific eQTL matched similar chromatin states in other cell types and tissues. ROADMAP chromatin states for each cell/tissue type were retrieved and plotted using the echoplot function in the echolocatoR R package suite [28]. Each cell type was accessed using Epigenome ID (EID) (S5 Table), as listed in the original study [12]. For example, chromatin states for “Primary hematopoietic stem cells” were retrieved using “E035” Epigenome ID.

## Results

### Statistical fine-mapping

In this study, we employed the SUSIE fine-mapping approach to prioritize causal SNPs within 64 psoriasis risk loci (out of a total of 67) previously reported in [15] (S1 Table). These risk loci were previously analyzed in [8] using meta-analysis and functional fine-mapping. We start by highlighting commonly encountered outcomes in statistical fine-mapping to make several points essential for practice. First, SNPs in strong LD to the lead SNP can sometimes be causal variants. Hence, focus on the lead SNP for functional study, as was done previously [33], can be potentially misleading if not supported by statistical fine-mapping. Secondly, strong LD in some risk loci makes statistical fine-mapping inefficient, and functional annotations or experiments are needed to prioritize functional SNPs.

**Fig 1:**
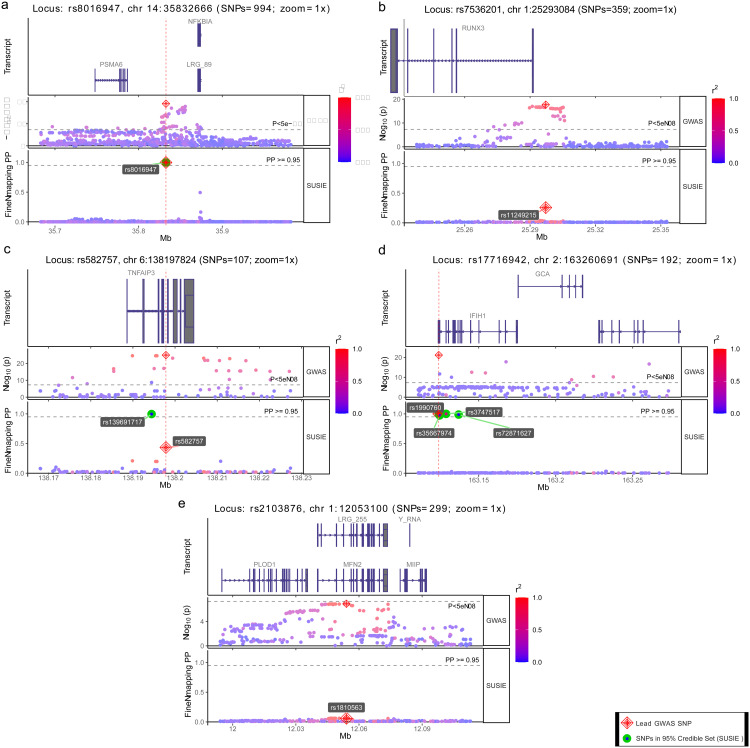
Five types of statistical fine-mapping outcomes. Figure panels a, b, c, d, and e depict annotation tracks for five psoriasis risk loci. For each risk locus, the upper track depicts the gene transcripts in the locus, the middle ‘GWAS’ track depicts log-transformed GWAS p-values for SNPs, and the bottom ‘SUSIE’ track represents fine-mapping results with posterior probabilities. The green circle indicates SNPs within 95% SUSIE Credible Set, i.e., predicted causal variants with strong support (PP ≥ 0.95). Red diamond indicates the lead GWAS SNP. **(A)** Statistical fine-mapping outcome: the GWAS lead SNP was prioritized as the most likely causal variant with strong evidence (PP ≥ 0.95). **(B)** The top-prioritized causal variant had only marginally higher PP than nearby SNPs. **(C)** Non-lead SNP had strong evidence of being causal (PP ≥ 0.95). **(D)** Multiple SNPs had strong evidence (PP ≥ 0.95) of being causal. **(E)** Strong LD rendered multiple causal SNP configurations equally possible, each with weak support. Statistical fine-mapping was ineffective.

We begin by demonstrating an example in which statistical fine-mapping was sufficient to prioritize the most likely causal variant with strong evidence, i.e., with a high posterior probability of being causal (PP ≥ 0.95; from here onward, posterior probability (PP) or probability). In Fig 1A, the lead SNP rs8016947 (indicated by a red diamond) has multiple adjacent variants with comparable p-values (“GWAS track”, Fig 1A). In this psoriasis locus, statistical fine-mapping (“SUSIE track”, Fig 1A) prioritized the lead SNP rs8016947 as the most likely (indicated by a red-green circle) causal variant (PP ≥ 0.95). This kind of desired fine-mapping outcome, however, represents a rare example (~9%, 6 out of 64 loci), in which statistical fine-mapping alone was sufficient to prioritize the causal variant with strong evidence (high PP ≥ 0.95). More often (~36%, 23 out of 64 loci), however, the degree of certainty in fine-mapping, as indicated by the posterior probability, is relatively low (well below 0.95). Usually, the top-prioritized SNP has only marginally higher PP than nearby SNPs (Fig 1B). For psoriasis, we inferred 23 such risk loci, where lead SNPs scored somewhat stronger evidence than nearby SNPs, but had posterior probabilities well below 0.95. For instance, in Fig 1B, the ‘GWAS’ track highlights a lead SNP, rs11249215, in the previously established risk locus with index SNP rs7536201. The lead SNP rs11249215 (red diamond), which falls outside the *RUNX3* coding sequence, is flanked by dozens of SNPs in strong LD (r^2^ ≥ 0.8) that have comparable p-values. Our statistical fine-mapping with SUSIE (Fig 1B, ‘SUSIE’ track) suggested that this non-coding lead SNP rs11249215 had only marginally higher PP (PP = 0.257) than flanking SNPs (PP varied from 0.03 to 0.05).

P-values reported by GWAS measure the probability of chance finding, but not the likelihood of causality. For that reason, the lead SNP with the lowest p-value does not necessarily represent the causal SNP. Yet, for psoriasis, earlier studies took lead SNPs from GWAS to design functional follow-up analyses [33]. Our statistical fine-mapping identified 7 psoriasis risk loci (7 out of 64), with non-lead SNPs achieving probabilities comparable to or higher than those of the lead SNPs. For example, Fig 1C illustrates GWAS SNPs in the rs582757 locus, scattered around the *TNFAIP3* gene transcript. There were multiple genome-wide significant SNPs in high LD, with the lead SNP rs582757 (“GWAS track”, Fig 1C). According to our fine-mapping analysis, one of the non-lead SNPs, rs139691717 (indicated by a green circle), has strong evidence of being causal (PP = 0.996). In contrast, the lead SNP, rs582757, had much less support (PP = 0.433) for being causal. We also encountered four loci (4 out of 65) where the lead SNP and one or more nearby SNPs were jointly inferred to be causal. For example, in two cases, the lead SNP and three nearby SNPs all had strong evidence (PP ≥ 0.95) of being causal (‘SUSIE’ track, Fig 1D), while in two other loci, the lead SNP and nearby SNPs all had identical but low PP (PP < 0.95) (Not shown).

Finally, strong linkage disequilibrium often (~37% of loci, 24 out of 65) renders fine-mapping ineffective. With strong LD (r2 ≥ 0.8), many causal SNP configurations are equally possible, and many SNPs have individually small posterior probabilities. Fig 1E showcases this frequent fine-mapping outcome. In this example, the lead SNP, rs1810563, in the previously established risk locus (with index SNP rs2103876), received weak evidence (PP well below 0.2) of being causal, like other linked SNPs in the vicinity (Fig 1E).

### Colocalizing GWAS SNPs with eQTLs from 48 GTEx tissues and 23 immune cells

**Fig 2:**
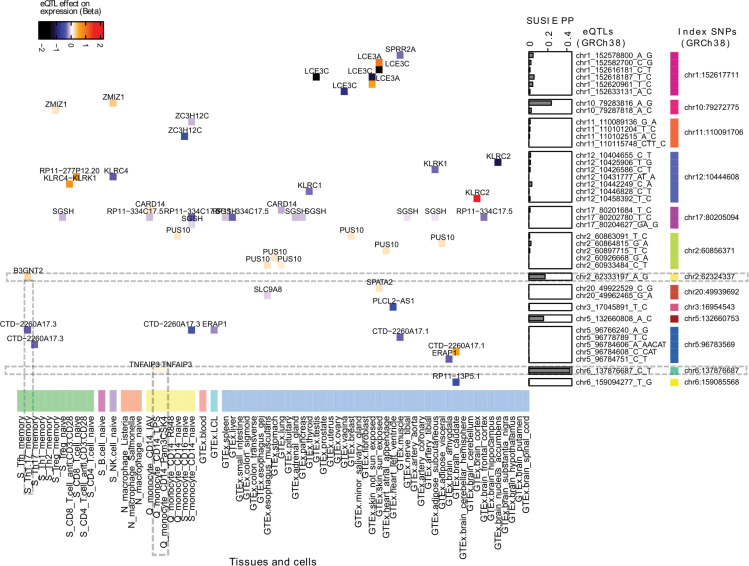
Expression QTLs colocalized with GWAS SNPs. Heatmap cells, the color-filled rectangles, represent colocalized eQTLs with effector gene names. Each eQTL has a line with the genomic position (GRCh38) and a column indicating the cell/tissue in which it was identified in the original eQTL mapping study. For each colocalized eQTL, the rectangle color indicates whether its effect allele increases (from white to red) or decreases (from white to black) the effector gene expression. For instance, in the upper left corner, C allele for the eQTL chr10_79287818_A_C increases ZMIZ gene expression in T regulatory cells with memory phenotype (‘S_Treg_memory’). Note that this eQTL chr10_79287818_A_C has another nearby eQTL chr10_79283816_A_G that also affects ZMIZ gene expression, but in naive NK cells ‘S_NK.cell_naive’, and this eQTL has a higher posterior probability of being causal (PP) based on SUSIE fine-mapping. Both eQTLs were in strong LD with the index SNP chr10:79272775.

Like an earlier study [8], our statistical fine-mapping with SUSIE prioritized multiple promising causal SNPs within 64 risk loci (established in [15]) (S1 Table). Most risk loci are presumed to influence disease by affecting gene expression [34]. To link our prioritized SNPs with nearby gene expression, we performed colocalization analysis with eQTL markers from different tissues. In total, we run colocalization for cis-acting eQTLs affecting gene expression in 48 human body tissues/cells [10,18] and 23 immune cell types and states (naïve, activated, and memory cells) [18–22] (S2 Table). Altogether, we colocalized 194 eQTLs with PP4 ≥ 0.8 at a 10% false discovery rate (FDR) across 69 (out of 71 analyzed) tissues and cell types (S3 Table). Of these, 39 were in high LD (r² ≥ 0.8) (Fig 2) (S4 Table), with 13 index SNPs in the previously established 64 risk loci (S1 Table).

### Prioritized causal SNP with putative regulatory function in effector/memory T cells

We used SUSIE-computed posterior probabilities to determine if any of the 39 eQTLs showed evidence of being causal within the 13 risk loci. Most of the 39 eQTLs (within 9 out of the total 13 risk loci) lacked strong evidence of being causal. None of them stood out with a high probability (PP ≥ 0.95) to prioritize. Instead, most of them, on average, had relatively small PP (PP ≤ 0.1) (See “SUSIE PP” bar plots in Fig 2). Nevertheless, our extended panel of immune cells (altogether 23) with different phenotypes (naive, activated, memory) allowed us to discover and prioritize two likely functional SNPs among probable causal SNPs within two risk loci (with index SNP rs10865331 and rs582757, respectively) (S1 Table). These putative functional SNPs, the two eQTLs (indicated by horizontal dashed lines in Fig 2), could have been missed if we had analyzed only GTEx tissues, as many other studies do. Furthermore, we highlighted these two eQTLs because experimental follow-up would be easier for them, as they function in specific cell types. Also, it would be easier to identify supporting (but independent) functional annotations in particular cell types, then test their absence in other cells and tissues. Taking into account these practical considerations, we focused on one of the discovered eQTLs, rs4672505 (indicated as chr2_62333197_A_G and marked by a horizontal dashed line in Fig 2), which was present only in memory Th1/17 cells (highlighted by a vertical dashed line in Fig 2). Since this eQTL was absent in other T cell types and GTEx tissues, we reasoned that its function must be confined to memory Th1/17 cells. These memory Th1/17 cells (denoted as ‘S_Th1.17_memory’ in Fig 2) were defined by a surface marker set (CD3 + /CD4 + /CD25low/CD45RA-/CD127high/CCR3-/CCR4 + /CCR6+) in the original study [19] and represent a mixture of CD + T helper 1 and T helper 17 cells with memory phenotype (Th1 and Th17 cells from here onward).

### Active chromatin state specific to effector/memory T cells

**Fig 3:**
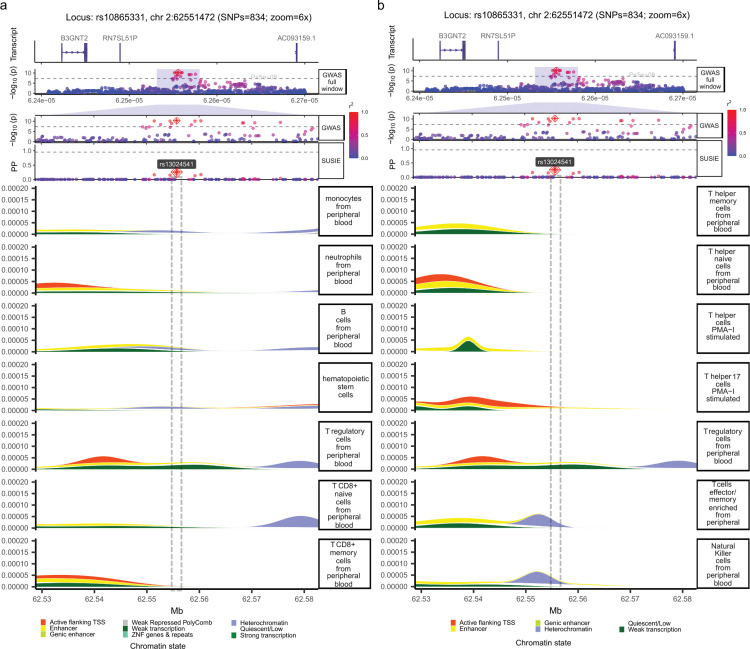
Chromatin states from different cell types combined with fine-mapping results for the risk locus rs10865331. Our target eQTL, rs4672505, is very close to the lead SNP, rs13024541, at the centre of the psoriasis risk locus, with the index SNP rs10865331. Figure tracks from top to bottom depict gene transcript annotations ‘Transcript’ for the risk locus, GWAS results, SUSIE-based statistical fine-mapping, and chromatin states from different cell types. **(A)** Chromatin states in immune cells, except CD4 + T helper cells. **(B)** Chromatin states in different CD4 + T helper cell subtypes and “NK cells”.

The memory phenotype of these Th1 and Th17 cells indicates that these T cells had previously encountered cognate antigen, i.e., were activated by specific foreign (or self) antigens via TCR receptor [35]. We therefore hypothesized that our eQTL site, rs4672505, likely overlaps a regulatory element that opens as naive CD4 + T cells transition into a memory state by remodeling their chromatin to an active state. Our hypothesis assumed that all other cells and tissues must lack this active chromatin state at our eQTL site. Moreover, this eQTL site must also be repressed at all stages of T cell ontogeny, i.e., before naive T cells turn into memory cells. To test this working hypothesis, we screened for active (e.g., active transcription start sites (TSSs) and enhancers) and inactive chromatin states (e.g., repressed Polycomb) across different tissues and immune cells available in the ROADMAP dataset (S5 Table) [12]. Specifically, we examined chromatin peaks around our eQTL site rs4672505 and for the entire risk locus. The ROADMAP dataset allowed us to screen chromatin marks in all the immune cells representing major steps in the immune cell ontogeny: starting with common hematopoietic stem cells, and ending with major derived cell fates, such as myeloid cells (monocytes and neutrophils), lymphoid cells (T cells, B cells), and Natural Killer cells. Our search did not find any clear chromatin peaks indicating active chromatin states at the queried eQTL site, neither in hematopoietic stem cells nor among major derived branches, such as myeloid cells (monocytes and neutrophils), and most lymphoid cells (B-cells and some T cells, different from CD4 + helper cells (Fig 3A). Reassuringly, we also did not find active chromatin states in non-immune tissues (S1 Fig).

Consistent with our eQTL-based hypothesis (eQTL unique to memory CD4 + T cells, highlighted in Fig 2), we identified clear cell-specific chromatin peaks at our focal eQTL rs4672505 only in memory effector T cells (Fig 3B). We observed two overlapping chromatin states: one linked to an enhancer (active state) and another associated with a heterochromatin (inactive) mark (Enhancer peak is behind heterochromatin peak in Fig 3B, see also S2 Fig). Similar chromatin peaks were also found in Natural Killer cells (NK cells) (Fig 3B). However, these peaks cannot be reliably attributed to NK cells due to uncertainty about cell type purity. In the ROADMAP project, NK cells were defined by gating CD56-positive cells (see the source data link in S6 Table) [12]. It is known that the CD56 marker is expressed by various immune cells, including alpha-beta T cells, gamma-delta T cells, dendritic cells, and monocytes [36]. Therefore, the chromatin marks observed in the so-called “NK cells” could originate from any of these CD56-positive cell types. We conclude that the active and inactive chromatin marks at our eQTL site rs4672505 can only be definitively attributed to memory T cells, which were defined as ‘CD4+_CD25int_CD127+_Tmem_Primary_Cells’ in the ROADMAP study. Finally, several T cell subtypes (T CD8 + , T helper, T regulatory) exhibit transcription-associated active chromatin states (‘Active flanking TSS’) around the *B3GNT2* gene, the effector gene for our eQTL (Fig 3). This widespread transcriptional activity among T cells supports the idea that transcription-associated states tend to be more constitutive across related cell types. In contrast, enhancer-associated states identified in effector memory T cells are typically highly tissue-specific [12].

In summary, our results suggest that observed immune-cell-specific eQTL from the eQTL Catalogue and chromatin marks from the ROADMAP provide two independent lines of evidence that our fine-mapped SNP, rs4672505, represents a regulatory variant at genomic position GRCh38.2:62333197:A/G. Given eQTL findings, allele G is associated with the higher expression of the *B3GNT2* gene in memory in Th1/17 cells (Fig 2). In contrast, the alternative A allele is associated with lower *B3GNT2* expression and increased psoriasis risk. According to the literature, B3GNT2 protein deficiency reduces post-translational modification of the T cell CD28 co-receptor, leading to stronger T cell activation.

## Discussion

### Statistical fine-mapping alone is not enough to resolve promising causal variants

We show that statistical fine-mapping alone yields only a few clear candidates for experimental follow-up, consistent with current field observations. According to the literature, only a few risk loci for complex diseases have been characterized mechanistically [9,37]. For example, for psoriasis, the latest fine-mapping study prioritized 14 candidate variants with highly probable regulatory functions [8]. Dand et al. reported a list of candidate causal SNPs, LD blocks, and multiple genes falling within 109 independent association signals (summarized in Table 10 of the original study) [8]. Reported credible sets with causal SNPs had an average size of 74 kb and contained dozens of highly correlated SNPs, in strong LD. Poor fine-mapping resolution is explained by strong linkage disequilibrium, rooted in the unique demographic history of extant human populations [38]. Namely, most human populations stem from a recent (~50,000 years ago) strong bottleneck event from Africa [39].

In our study, for most risk loci, statistical fine-mapping was also insufficient to narrow down individual candidates for causal variants (Fig 1). For example, for our focal risk locus tagged by index SNP rs10865331 (“GWAS full window” and “GWAS” tracks in Fig 3A), we observed several strongly linked candidate SNPs (rs13024541 with the highest PP) with comparable SUSIE PP (“SUSIE” track in Fig 3A), which hindered causal SNP prioritization. For the same risk locus, a previous study used genotypes from European and trans-ethnic cohorts and narrowed down (95% Bayesian Credible Interval) 9 and 11 SNPs, respectively [15].

Strong LD often prevents fine-mapping in the study population, which usually (~90%) consists of patients with European ancestry [40]. In such cases, one can achieve further progress by adding trans-ethnic cohorts, if available [15]. In practice, however, multi-ancestry GWAS data [41] are rare, and the application of multi-ancestry fine-mapping is rare. More often, further prioritization is achieved by functional annotation-informed fine-mapping [42]. i.e., by adding functional annotations.

### Systematic fine-mapping assisted by functional annotations

To prioritize causal variants among linked SNPs in our risk locus of interest (with index SNP rs10865331, defined as chr2:62551472 in [15], we added functional annotations. We sought SNPs with a function in immune cells relevant to psoriasis pathogenesis. By adding 23 immune cell types to the 49 GTEx tissues (S2 Table), we colocalized eQTL present only in memory Th1/17 cells. This helped us prioritize a potentially functional SNP with a regulatory effect on the nearby *B3GNT2* gene expression, a cell-specific effect detected so far only in memory Th1/17 cells. The affected *B3GNT2* gene was more distal to the eQTL (rs4672505) than other proximal transcripts annotated in this region (“Transcript” track in Fig 3A). Starting with the earliest post-GWAS studies, the *B3GNT2* gene was suspected as the effector gene based on the nearest-gene principle and the assumption that the index (leading) SNP [43,44] is causal. This gene was also nominated as an effector gene for psoriatic arthritis based on its proximity to the lead SNP [45]. Currently, it is widely accepted that hand-picking the nearest gene as an effector gene can be misleading unless supported by functionally informed fine-mapping [3]. Moreover, a leading SNP in the risk locus may not be the causal SNP, as we show in our Results section. Recently, the TWAS approach helped include the *B3GNT2* gene on the list of candidate effector genes for psoriasis [8]. Unfortunately, the TWAS approach has known pitfalls. This approach “does not allow for any conclusions to be drawn about the relationship of the phenotype with the gene expression” [46]. Finally, for our risk locus of interest (with index SNP rs10865331), a recent study inferred a different effector gene (than *B3GNT2)*, the *COMMD1* gene, approximately 435 kb upstream of the locus [33]. This study used a capture Hi-C (CHi-C) approach to find a long-range interaction between the index SNP rs10865331 and the *COMMD1*, but not *B3GNT2* [33]. This inference is problematic for two reasons. Firstly, index SNP rs10865331 was picked as a candidate causal variant without performing statistical fine-mapping. In our study, we deliberately reported SUSIE fine-mapping outcomes to emphasize that the lead (and index) SNP does not always represent the causal variant (Fig 1). This is also likely true for our focal risk locus (“SUSIE” track in Fig 3A), where a non-lead SNP, rs13024541, was a more promising candidate. Secondly, the authors did CHi-C interaction experiments using immortalized keratinocytes and CD8 + T cells [33]. Immortalized cell lines exhibit substantial differences in chromatin accessibility, nucleosome positioning, histone modifications, and DNA methylation compared with primary cells [47]. Therefore, capture Hi-C readouts reported in [33] may reflect an altered regulatory landscape in immortalized cell lines. It is not clear whether the observed long-range loops to the distal *COMMD1* gene [33] are present in the primary cells in the disease context. In the meantime, chromatin accessibility in primary cells supports our inference about the functional link to the *B3GNT2* gene. Namely, there is evidence for a functional link between rs4672505 and *B3GNT2* gene expression coming from primary cells from Ankylosing spondylitis patients, who share the same risk locus as psoriasis patients [48]. In ankylosing spondylitis patients, primary CD8 + T cells demonstrate chromatin interaction loops between the *B3GNT2* gene and rs4672505, which falls within an enhancer element. While this study supports our hypothesis about the enhancer element at the eQTL site (rs4672505), the identified loop was observed in CD8 + T cells but not in CD4 + T cells. Our inferences about the risk variant location and function agree with functional fine-mapping at the shared risk loci for ankylosing spondylitis, except that cell-specificity appears to differ. Thus, we have indirect support for our inference of a link between rs4672505 and *B3GNT2* gene expression. Still, most previous studies on psoriasis suggested effector genes for the analyzed risk locus (rs10865331) without following or omitting key steps required for systematic fine-mapping. None of the earlier studies on psoriasis simultaneously inferred the cell type associated with the disease SNP, the molecular mechanism (promoter, enhancer, or transcription factor (TF) binding site), or whether the risk allele increased or decreased effector gene expression.

### The place and timing of the putative causal SNP function

Our findings suggest that psoriasis patients who carry the risk allele at rs4672505 likely have lower *B3GNT2* expression in memory T helper cells. B3GNT2 encodes a Golgi-resident enzyme, Beta-1,3-N-acetylglucosaminyltransferase 2, which glycosylates cell surface glycoproteins on immune cells and thereby regulates immune cell signaling [49]. In CD4 + T helper cells, *B3GNT2* deficiency reduces glycosylation of the CD28 coreceptor and renders CD4 + T cells hyperreactive upon antigen-specific TCR stimulation. We, therefore, hypothesize that lower *B3GNT2* expression in risk allele carriers can result in reduced CD28 co-receptor decoration on memory T helper cells. The CD28 co-receptor provides the second important signal for T cell activation and proliferation. Namely, when naïve CD4 + T cells recognize their specific antigens via TCR, the “signal 1” they additionally need to receive “signal 2” on CD28 from antigen-presenting cells expressing B7. Typically, antigen-presenting cells need to encounter conserved microbial products to express B7 and thereby ensure that the presented antigen (signal 1) is associated with pathogen entry [50]. We found that a genetic risk allele increases the signal 2 in memory T helper cells, and they, like naïve T cells, also require CD28 co-stimulation upon re-exposure to their cognate antigen via their TCR receptor [51], the signal 1. These details are essential to understanding how the *B3GNT2-*related risk factor could be mechanistically involved in the pathogenesis. As mentioned above, this genetic risk factor can increase the sensitivity of CD + T cells to their cognate antigens via TCR. However, sensitivity to the cognate antigen (signal 1) is determined by low-glycosylated CD28, and the CD28 co-receptor receives “signal 2” only when APCs express B7 in the presence of conserved microbial products, which can be derived from pathogens (including vaccines) [52]. Taken together, we hypothesize that memory T helper cells in psoriasis patients are more sensitive to re-exposure to self-antigens in the context of infection or vaccination, consistent with known features of psoriasis. For instance, psoriasis often manifests, exacerbates, or reappears when patients encounter infections or get vaccinated [53–57]. Our findings are consistent with a growing body of evidence that causal variants for immune-mediated diseases can occur in dynamic regulatory elements that “stay quiescent” until immune cells activate, for example, in response to cell-specific environmental cues/triggers [58–60]. Thus, filling the missing link between disease risk loci and function would require not only more readouts (such as pQTLs, splice variants, chromatin marks, etc.) from the same tissues but also more experiments with disease-relevant tissues under disease-relevant physiological states.

### Implications for the follow-up experiments

Our findings offer a hypothesis for directing experimental research – information about the cell type and molecular phenotype to test in vitro or in vivo. Specifically, one must focus on primary Th1 and Th17 cells from patients, rather than on reprogrammed or otherwise manipulated cell cultures to recapitulate the unique chromatin landscape around the causal SNP. Cell populations from risk-allele carriers and non-carriers can be tested for *B3GNT2* gene expression and for polylactosamine decoration of CD28 receptors. The associated pathogenic phenotype, according to our hypothesis, must be increased sensitivity to activation by cognate antigen. For psoriasis, one can test one of the known protein epitopes [61]. If confirmed, polylactosamine decoration, memory T cells, or even infections in patients can be a potential therapeutic target.

## Conclusions

Statistical fine-mapping alone was insufficient to prioritize causal variants for most psoriasis risk loci due to strong linkage disequilibrium. Functionally informed fine-mapping with annotations from disease-relevant cell types (e.g., eQTL in memory Th1/Th17 cells) was crucial for prioritizing candidate SNPs (e.g., rs4672505) and their effector genes (e.g., B3GNT2). Our findings suggest that the risk allele at rs4672505 likely reduces B3GNT2 expression in memory T helper cells, leading to hyperreactivity via reduced CD28 glycosylation. We hypothesize that T cell hyperreactivity phenotypically manifests in response to danger signals, as CD28 co-signalling normally occurs after antigen-presenting cells encounter conserved microbial products.

## Supporting information

S1 Fig Chromatin states from different body tissues combined with fine-mapping results for the risk locus rs10865331. Our target eQTL, rs4672505, is very close to the lead SNP rs13024541 at the center of the psoriasis risk locus with index SNP rs10865331. Figure tracks from top to bottom depict gene transcript annotations ‘Transcript’ for the risk locus, GWAS results, SUSIE-based statistical fine-mapping, and chromatin states from different cell types.
(PDF)

S2 Fig Chromatin peaks in T effector/memory cells within the risk locus rs10865331. (PDF)

S1 Table Genomic coordinates for psoriasis risk loci. Psoriasis risk loci were defined based on index SNPs reported by [15], in Table S5 of the original study. Specifically, we used SNP IDs from the “ID” column.
(XLSX)

S2 Table Expression QTL datasets used for colocalization with psoriasis candidate risk SNPs. eQTL summary data for each tissue/cell type were retrieved from the eQTL Catalogue project (https://www.ebi.ac.uk/eqtl/). eQTL Catalogue stores uniformly processed gene expression and splicing QTLs from published studies.
(XLSX)

S3 Table Expression QTLs colocalized with psoriasis GWAS SNPs. Matrix columns correspond to eQTLs from different tissue/cell types. Matrix rows correspond to psoriasis GWAS SNPs that showed strong evidence (Posterior Probability, PP4 ≥ 0.8) for colocalization with a given eQTL at a given row. Each matrix cell contains a gene name for the affected gene and a Beta-value. Beta-value has a positive or negative value depending on whether the eQTL effect allele increases or decreases gene expression. For example, consider the cell entry, “*ZMIZ1* +0.290125”, at the intersection of the first line, “chr10_79283816_A_G”, and the column “Schmiedel_2018*NK-cell_naive_ge”. Here, the G allele for eQTL chr10_79283816_A_G increases the expression of the *ZMIZ1* gene in naive NK-cells (Schmiedel_2018*NK-cell_naive_ge) proportional to Beta = +0.290125 relative to the alternative A allele.
(XLSX)

S4 Table Colocalized expression QTLs in strong LD with psoriasis GWAS signals. Colocalized eQTLs were tested for linkage disequilibrium with index SNPs for known psoriasis risk loci using pairwise r2. Altogether 39 eQTLs were found to be in strong LD (r2 ≥ 0.8) and were retained for analyses.
(XLSX)

S5 Table Сhromatin state annotations from ROADMAP screened in this study. Cells and tissues for which chromatin peaks were screened using echoplot::plot_locus function in the echolocatoR package [28]. Each cell and tissue was retrieved using unique epigenome IDs.
(XLSX)

S6 Table Chromatin state annotations as presented in Fig 3. List of cells and tissues from ROADMAP presented in Fig 3.
(XLSX)
