# Supplementary material for: Psoriasis risk allele function in activated Th1/17 cells with “memory” to antigen exposure
Source: PLoS One. 2026 Mar 11;21(3):e0344675. doi: 10.1371/journal.pone.0344675 (PMC12978443; doi:10.1371/journal.pone.0344675)
Supplement: S1 Fig — Our target eQTL, rs4672505, is very close to the lead SNP rs13024541 at the center of the psoriasis risk locus with index SNP rs10865331. Figure tracks from top to bottom depict gene transcript annotations ‘Transcript’ for the risk locus, GWAS results, SUSIE-based statistical fine-mapping, and chromatin states from different cell types. (PDF) [file pone.0344675.s001.pdf]

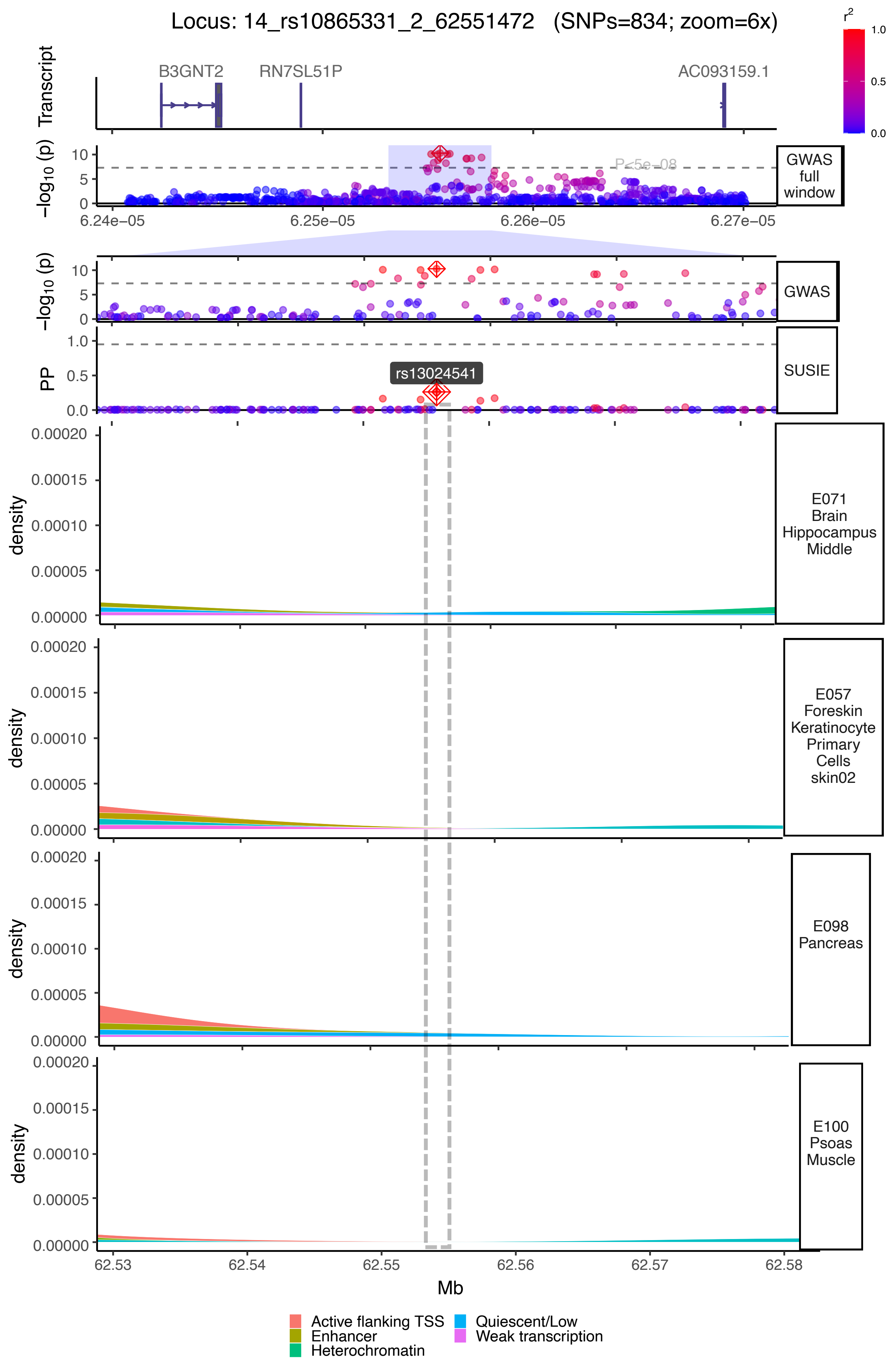

Locus: 14_rs10865331_2_62551472 (SNPs=834; zoom=6x)
Transcript
B3GNT2
RN7SL51P
AC093159.1
r2
1.0
0.5
0.0
−log10 (p)
10
5
0
P<5e−08
6.24e−05
6.25e−05
6.26e−05
6.27e−05
GWAS full window
GWAS
PP
1.0
0.5
0.0
rs13024541
SUSIE
density
0.00020
0.00015
0.00010
0.00005
0.00000
E071 Brain Hippocampus Middle
E057 Foreskin Keratinocyte Primary Cells skin02
E098 Pancreas
E100 Psoas Muscle
62.53
62.54
62.55
62.56
62.57
62.58
Mb
Active flanking TSS
Enhancer
Heterochromatin
Quiescent/Low
Weak transcription
