# Supplementary figures and images for: Psoriasis risk allele function in activated Th1/17 cells with “memory” to antigen exposure
Source: PLoS One. 2026 Mar 11;21(3):e0344675. doi: 10.1371/journal.pone.0344675 (PMC12978443; doi:10.1371/journal.pone.0344675)

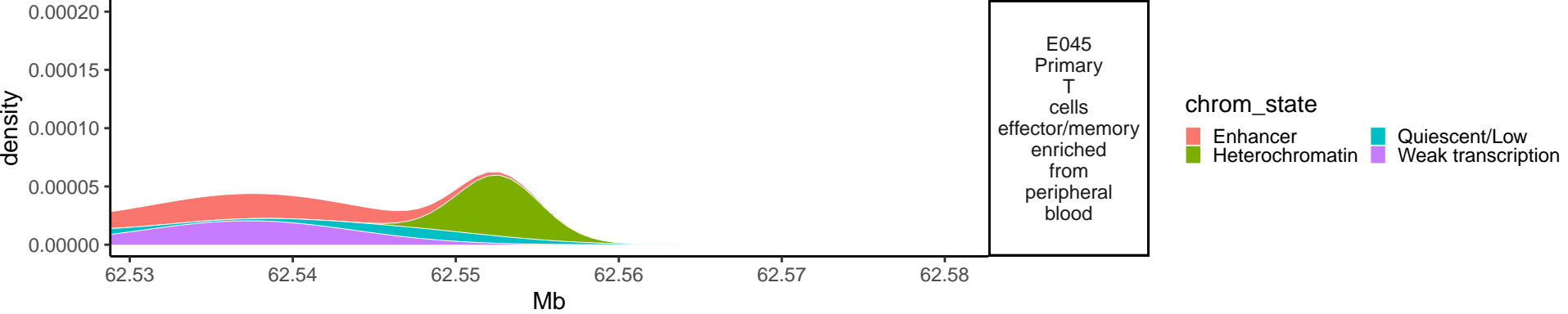

Supplement: S2 Fig — (PDF) [file pone.0344675.s002.pdf]
